# Host Genetics Predict Clinical Deterioration in HCV-Related Cirrhosis

**DOI:** 10.1371/journal.pone.0114747

**Published:** 2014-12-12

**Authors:** Lindsay Y. King, Kara B. Johnson, Hui Zheng, Lan Wei, Thomas Gudewicz, Yujin Hoshida, Kathleen E. Corey, Tokunbo Ajayi, Nneka Ufere, Thomas F. Baumert, Andrew T. Chan, Kenneth K. Tanabe, Bryan C. Fuchs, Raymond T. Chung

**Affiliations:** 1 Liver Center, Gastrointestinal Division, Massachusetts General Hospital, Boston, Massachusetts, United States of America; 2 Department of Medicine, Massachusetts General Hospital, Boston, Massachusetts, United States of America; 3 Department of Surgery, Massachusetts General Hospital, Boston, Massachusetts, United States of America; 4 Department of Pathology, Massachusetts General Hospital, Boston, Massachusetts, United States of America; 5 Biostatistics Center, Massachusetts General Hospital, Boston, Massachusetts, United States of America; 6 Harvard Medical School, Boston, Massachusetts, United States of America; 7 Department of Medicine, Icahn School of Medicine at Mount Sinai, New York, New York, United States of America; 8 Department of Medicine, North Shore Medical Center, Salem, Massachusetts, United States of America; 9 Inserm U1110, University of Strasbourg, Strasbourg, France; University of Modena & Reggio Emilia, Italy

## Abstract

Single nucleotide polymorphisms (SNPs) in the epidermal growth factor (*EGF*, rs4444903), patatin-like phospholipase domain-containing protein 3 (*PNPLA3*, rs738409) genes, and near the interleukin-28B (*IL28B*, rs12979860) gene are linked to treatment response, fibrosis, and hepatocellular carcinoma (HCC) in chronic hepatitis C. Whether these SNPs independently or in combination predict clinical deterioration in hepatitis C virus (HCV)-related cirrhosis is unknown. We genotyped SNPs in *EGF, PNPLA3*, and *IL28B* from liver tissue from 169 patients with biopsy-proven HCV cirrhosis. We estimated risk of clinical deterioration, defined as development of ascites, encephalopathy, variceal hemorrhage, HCC, or liver-related death using Cox proportional hazards modeling. During a median follow-up of 6.6 years, 66 of 169 patients experienced clinical deterioration. *EGF* non-AA, *PNPLA3* non-CC, and *IL28B* non-CC genotypes were each associated with increased risk of clinical deterioration in age, sex, and race-adjusted analysis. Only *EGF* non-AA genotype was independently associated with increased risk of clinical deterioration (hazard ratio [HR] 2.87; 95% confidence interval [CI] 1.31–6.25) after additionally adjusting for bilirubin, albumin, and platelets. Compared to subjects who had 0–1 unfavorable genotypes, the HR for clinical deterioration was 1.79 (95%CI 0.96–3.35) for 2 unfavorable genotypes and 4.03 (95%CI 2.13–7.62) for unfavorable genotypes for all three loci (P_trend_<0.0001). In conclusion, among HCV cirrhotics, *EGF* non-AA genotype is independently associated with increased risk for clinical deterioration. Specific *PNPLA3* and *IL28B* genotypes also appear to be associated with clinical deterioration. These SNPs have potential to identify patients with HCV-related cirrhosis who require more intensive monitoring for decompensation or future therapies preventing disease progression.

## Introduction

Chronic hepatitis C (CHC) is the most common cause of liver-related death and liver transplantation in the United States [1]. The rate of progression of hepatitis C virus (HCV) infection is variable, likely due to a combination of host genetic and environmental factors. At least 20% of patients with CHC develop cirrhosis over a twenty-year period [2]. Once cirrhosis is established, patients are at risk for hepatocellular carcinoma (HCC) and decompensation, characterized by ascites, variceal hemorrhage, or hepatic encephalopathy (HE), and survival decreases from a median of 12 years to 2 years [3].

Attempts have been made to develop risk scores to predict the risk of disease progression for individual patients. Such scores have incorporated both clinical variables and genetic data [4]. The possibility of tailoring clinical management to genetic data is exciting, but the discovery of ever-increasing numbers of single nucleotide polymorphisms (SNPs) associated with liver disease mandates careful selection of polymorphisms that have independent predictive value for relevant outcomes. One of the genetic variations for which there is compelling evidence is the rs12979860 SNP near the interleukin-28B (*IL28B*) locus. The CC *IL28B* genotype is associated with spontaneous clearance of HCV and predicts interferon and ribavirin treatment response [5], [6]. However, data regarding an independent association between *IL28B* genotype and disease course are conflicting [7]–[9]. The epidermal growth factor (*EGF*) gene polymorphism rs4444903 has been associated with EGF levels [10], HCC [10], [11] and fibrosis [12]. Last, while the patatin-like phospholipase domain-containing protein 3 (*PNPLA3*) SNP rs738409 has mainly been studied in nonalcoholic fatty liver disease (NAFLD) [13], studies in patients with CHC have shown an association with steatosis, fibrosis [14], [15], and HCC [15], [16], although data are conflicting [17].

These data suggest that these SNPs may be useful for the prediction of the natural history of CHC. However, no study has evaluated the influence of *IL28B, EGF*, and *PNPLA3* genotypes on the natural history of HCV-related cirrhosis or examined these SNPs in the same population. We therefore sought to evaluate the association between these SNPs and clinical deterioration in a cohort of patients with HCV-related cirrhosis.

## Materials and Methods

### Cohort assembly

**Figure 1:**
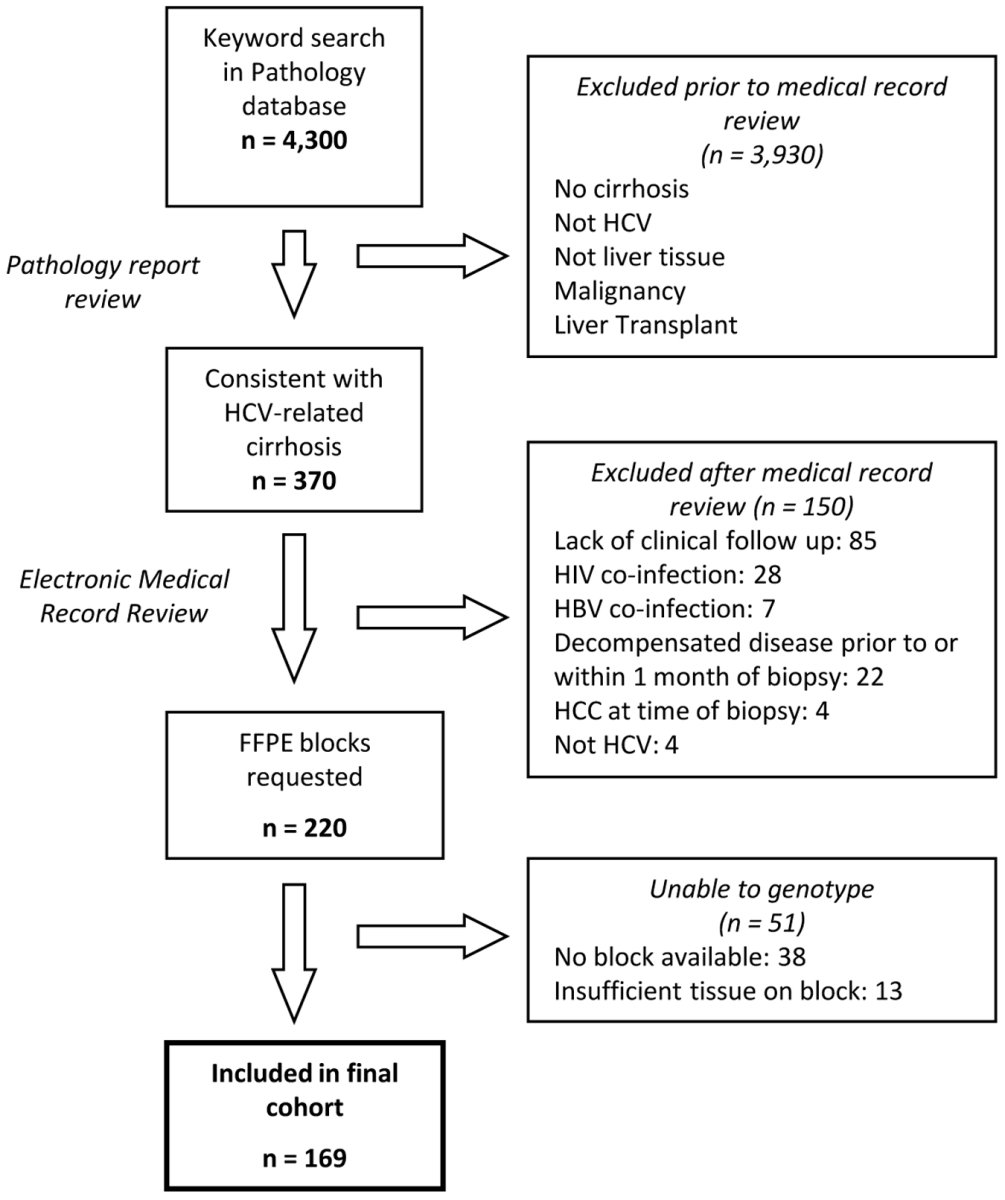
Flow chart for identification of the cohort. The following keywords were used in the pathology database search: HCV, HBV, NAFLD, NASH, and hepatitis. HCV: hepatitis C virus; HBV: hepatitis B virus; HIV: human immunodeficiency virus; HCC: hepatocellular carcinoma; FFPE: formalin-fixed, paraffin-embedded;

The patient cohort was identified from pathology reports at Massachusetts General Hospital. A natural language search for biopsies performed between 1990 and 2007 was previously performed for the keywords: HCV, HBV, NAFLD, NASH, and hepatitis. Pathology reports were reviewed to identify patients whose biopsies were consistent with HCV-related cirrhosis. Inclusion criteria were age ≥18 years at time of biopsy, positive HCV antibody or HCV RNA, and presence of cirrhosis (Ishak stage 5 or 6/6 or Metavir stage 4/4). Exclusion criteria included co-infection with human immunodeficiency virus or hepatitis B virus (HBV), liver transplantation, ascites, variceal hemorrhage, HE, or HCC prior to or within one month of the biopsy, and lack of follow-up data following the biopsy. The electronic medical records of patients identified by the pathology database search as having biopsies consistent with HCV-related cirrhosis were manually reviewed by two independent reviewers. The keyword search of the pathology database yielded 370 patients whose reports were consistent with a diagnosis of HCV-related cirrhosis. After review of the medical record, 220 patients were eligible. Formalin-fixed, paraffin-embedded (FFPE) blocks were not available for 38 patients, and genotyping could not be performed on 13 patients. Thus, the final cohort included 169 patients (Fig. 1). Nine patients overlapped with the cohort in which we previously identified the association between *EGF* genotype and HCC [10]. This study was approved by the Partners Human Research Committee. The Committee waived the need for written, informed consent for this retrospective study. All data was analyzed anonymously.

### Ascertainment of Outcomes

The follow-up period for each patient was defined as the date of the index biopsy until the occurrence of the first episode of clinical deterioration, death, loss to follow-up, or December 31, 2012, whichever came first. Clinical deterioration was defined as the development of ascites, HE, variceal hemorrhage, HCC, or liver-related death. Outcomes were identified by manual chart review performed by two independent reviewers. The primary outcome was the time to the first episode of clinical deterioration after the index biopsy.

### Other covariates

We collected the following baseline variables from the electronic medical record: age, gender, race, medical history, body mass index, alanine aminotransferase (ALT), aspartate aminotransferase (AST), total bilirubin, albumin, platelet count, creatinine, international normalized ratio [18] (INR), prothrombin time, HCV genotype and HCV RNA, alcohol use, and smoking history. For all baseline laboratory values, we used the value determined at the time of the index biopsy. If no value was available at that time, we used the value collected closest to the biopsy within the year prior to the biopsy. Heavy alcohol use was defined as documentation of alcohol abuse or dependence, a history of substance abuse counseling, or consumption >14 drinks per week by males or >7 drinks per week by females. Diabetes was defined as a history of diabetes mellitus in the medical record or the use of diabetic medications. The number of subjects with incomplete data was 48 for HCV RNA; 39 for creatinine; 22 for HCV genotype; 16 for either prothrombin time or INR; 13 for smoking status; 13 for ALT, AST, albumin, total bilirubin; 9 for platelets; and 8 for alcohol use.

### Genotyping

DNA was extracted from FFPE liver tissue (Five 10 µm sections from each patient) using the QiaAMP FFPE Tissue Kit (Qiagen Inc, Valencia, CA). Genotyping was performed on 5 ng of DNA using the 7900HT Fast Real-Time PCR System with commercial TaqMan SNP Genotyping Assays for *IL28B* rs12979860, *EGF* rs4444903, and *PNPLA3* rs738409 (Life Technologies, Grand Island, NY) according to the manufacturer's instructions. Genotypes were assigned using Sequence Detection System (SDS 2.4) software with manual review by two independent investigators, blinded to subject phenotype.

### Statistical analysis

We calculated the person-years of follow-up for each individual from the date of the biopsy to the development of the first episode of clinical deterioration, date of death, or end of follow-up, whichever came first. Kaplan-Meier method was used to analyze the time to clinical deterioration. The log rank test was used for comparison between genotypes. The crude and adjusted hazard ratios (HRs) and 95% confidence intervals (CIs) for the effect of the SNPs on the rate of clinical deterioration were estimated with Cox proportional hazards regression models. The proportional hazard assumption was checked via the statistical significance of the interaction between log (follow-up time) and genotype. For our primary analysis, we examined each SNP in an age, sex, and race-adjusted model. In a secondary analysis, we also examined the association of each SNP in a multivariable model additionally adjusting for established predictors of clinical deterioration including baseline albumin, platelets, and total bilirubin [3], [19]. Lastly, we included all 3 SNPs in a combined genotype model in which we compared subjects who had an unfavorable genotype for all 3 SNPs to subjects who had two, one or zero unfavorable genotypes.

We performed several sensitivity analyses. First, we performed an analysis excluding HCC and HCC-related death as a first outcome. For this analysis, we calculated the person-years of follow-up for each individual from the date of biopsy to the development of the first episode of ascites, variceal hemorrhage, HE, date of death, or end of follow-up, whichever came first. Second, we performed an analysis incorporating sustained virologic response (SVR) at any point prior to the censor date into the model given the known association between *IL28B* genotype and SVR. Third, we performed an analysis excluding the one subject who had an outcome within 6 months of the index biopsy. Finally, we performed an analysis excluding subjects who were Child-Pugh Class B or missing a Child-Pugh score because of missing laboratory data. A 2-tailed *P*-value <0.05 was considered statistically significant. SAS (Cary, NC) version 9.3 was used for statistical analyses.

## Results

Among the 169 patients with biopsy-proven HCV-related cirrhosis, the baseline demographic and clinical data according to *EGF*, *IL28B*, and *PNPLA3* genotypes are shown in Table 1. The mean age of the cohort was 50±9 years. The cohort was predominantly Caucasian (84%), male (74%) and had Child-Pugh Class A (93%) or Class B (7%) cirrhosis. After a median follow-up of 6.6 years (IQR: 4.7–9.2 years), 66 (39%) patients developed at least one episode of clinical deterioration. Outcomes included death related to portopulmonary hypertension, n = 1; ascites, n = 18; variceal hemorrhage, n = 13; HE n = 7; HCC, n = 18; ascites and HE, n = 7; variceal hemorrhage and HE, n = 1; and ascites and variceal hemorrhage, n = 1.

**Table 1:** Baseline Characteristics of all patients and stratified by genotype.

	Entire cohort	*EGF* AA	*EGF* AG/GG	*IL28B* CC	*IL28B* CT/TT	*PNPLA3* CC	*PNPLA3* CG/GG
Number	169	45	82/42	66	75/28	102	51/16
Age, years	50.1(9.3)	51.6(7.6)	49.6(9.8)	51.2(9.8)	49.4(8.6)	50.4(8.8)	49.6(10.1)
Male, %	74	69	76	68	78	72	78
Race							
White, %	84	89	82	91	80	85	82
Black, %	5	4	6	2	8	8	2
Hispanic,%	7	2	8	3	9	2	13
Other, %	4	4	4	4	4	5	3
Ever smoker, %	63	53	67	56	68	65	61
History of heavy alcohol use, %	51	53	51	42	57	52	51
Diabetes Mellitus, %	13	9	15	6	17	13	13
HCV RNA >500,000, IU/mL, %	47	53	44	53	43	45	49
Genotype 1, %	63	64	62	55	68	58	70
AST, U/L	115.3(73.8)	110.8(88.1)	117.0(67.8)	103.4(64.6)	122.5(78.2)	106.3(59.1)	128.7(90.4)
ALT, U/L	139.4(113.0)	134.1(111.0)	131.5(114.1)	142.4(118.6)	137.6(109.9)	129.1(98.5)	154.8(130.7)
Albumin, g/dL	3.8(0.5)	3.8(0.4)	3.8(0.6)	3.9(0.5)	3.7(0.5)	3.8(0.5)	3.7(0.5)
Creatinine, mg/dL	0.9(0.5)	1.0(0.8)	0.9(0.2)	0.9(0.2)	0.9(0.5)	1.0(0.6)	0.9(0.2)
Total Bilirubin, mg/dL	0.9(1.1)	0.7(0.4)	0.9(1.2)	0.66(0.3)	1.0(1.3)	0.9(1.3)	0.8(0.5)
Platelets ×1000/mm^3^	152.4(59.4)	170.0(49.5)	145.8(61.7)	161.3(59.8)	146.6(58.8)	156.1(60.8)	146.5(57.1)

	Entire cohort	*EGF* AA	*EGF* AG/GG	*IL28B* CC	*IL28B* CT/TT	*PNPLA3* CC	*PNPLA3* CG/GG
Child-Pugh Class							
A, %	82	91	78	82	82	81	82
B, %	7	2	8	2	10	7	6
Unknown, %	12	7	14	17	9	12	12

Continuous Variables are presented as mean (SD).

EGF: Epidermal Growth Factor; IL28B: interleukin-28B; PNPLA3: patatin-like phospholipase domain-containing protein 3; HCV: hepatitis C virus; AST: aspartate aminotransferase; ALT: alanine aminotransferase.

**Figure 2:**
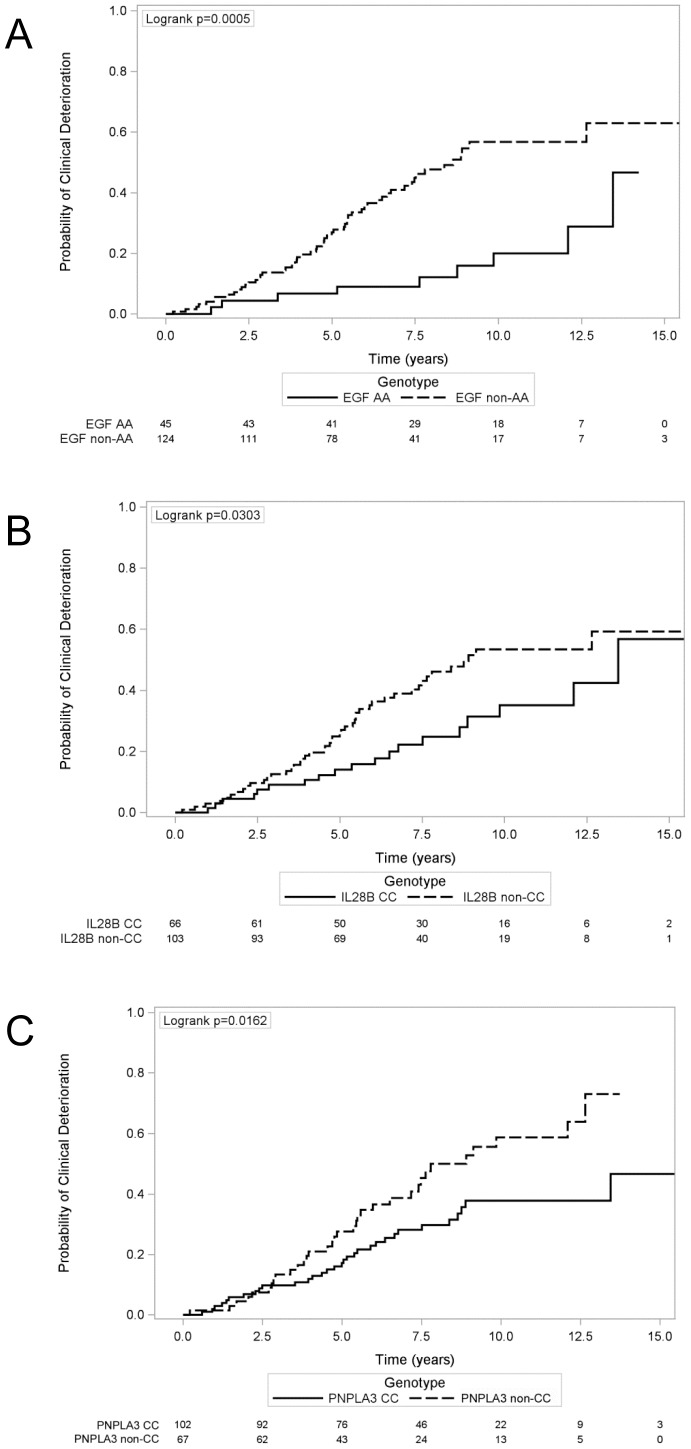
Kaplan-Meier analysis of clinical deterioration by genotype. A. Kaplan-Meier analysis of the time to first episode of ascites, variceal hemorrhage, hepatic encephalopathy, hepatocellular carcinoma, or liver-related death stratified by *EGF* genotype. B. Kaplan-Meier analysis of time to first episode of ascites, variceal hemorrhage, hepatic encephalopathy, hepatocellular carcinoma, or liver-related death stratified by *IL28B* genotype. C. Kaplan-Meier analysis of time to first episode of ascites, variceal hemorrhage, hepatic encephalopathy, hepatocellular carcinoma, or liver-related death stratified by *PNPLA3* genotype. EGF: Epidermal Growth Factor; IL28B: Interleukin-28B; PNPLA3: patatin-like phospholipase domain-containing protein 3.

To evaluate the association of genotype independent of other clinical factors, we used multivariable models adjusted for age, sex, and race (Table 2; Fig. 2). Compared with AA genotype, *EGF* non-AA genotype was associated with an age, sex, and race-adjusted HR of 3.20 (95% CI 1.57–6.52; p = 0.001) for clinical deterioration. Compared with CC genotype, *IL28B* non-CC genotype was associated with an age, sex, and race-adjusted HR of 1.78 (95%CI 1.03–3.06; p = 0.04) for clinical deterioration. Compared with CC genotype, *PNPLA3* non-CC genotype was associated with an age, sex, race-adjusted HR of 1.79 (95%CI 1.10–2.90; p = 0.02) for clinical deterioration.

**Table 2:** Cox proportional Hazards Model for Clinical Deterioration.

Genotype	Cases/Person-Years	Age, sex, race- Adjusted Hazard Ratio (95%CI)	P value	Multivariable Adjusted Hazard Ratio* (95% CI)	P value
*EGF* AA	9/390	1.00		1.00	
*EGF* non-AA	57/812	3.20 (1.57–6.52)	0.001	2.87 (1.31–6.25)	0.008
*IL28B* CC	19/506	1.00		1.00	
*IL28B* non-CC	47/697	1.78 (1.03–3.06)	0.04	1.38 (0.71–2.68)	0.34
*PNPLA3* CC	32/752	1.00		1.00	
*PNPLA3* non-CC	34/451	1.79 (1.10–2.90)	0.02	1.45 (0.85–2.47)	0.17

*Adjusted for age, sex, race, and baseline total bilirubin, albumin, and platelets.

EGF: Epidermal Growth Factor; IL28B: interleukin-28B; PNPLA3: patatin-like phospholipase domain-containing protein 3; CI: confidence interval.

In multivariable models with age, race, sex and established predictors of disease progression including albumin, platelets, and total bilirubin, *EGF* non-AA genotype remained an independent predictor of increased risk of clinical deterioration, multivariable-adjusted HR = 2.87 (95% CI 1.31–6.25; p = 0.008) (Table 2). In contrast, there was no longer a significant association between *IL28B* genotype (multivariable-adjusted HR 1.38; 95%CI 0.71–2.68; p = 0.34 for non-CC compared with CC) and *PNPLA3* genotype (multivariable HR 1.45; 95%CI 0.85–2.47, p = 0.17 for non-CC compared with CC) and clinical deterioration.

When all three SNPs were analyzed together, the presence of 3 unfavorable genotypes (*EGF* non-AA, *IL28B* non-CC, and *PNPLA3* non-CC, HR 4.03; 95%CI 2.13–7.62) and 2 unfavorable genotypes (HR 1.79; 95%CI 0.96–3.35) were associated with a significantly increased risk of clinical deterioration compared to the presence of one or zero unfavorable genotypes (P_linear_ _trend_<0.0001) (Table 3).

**Table 3:** Combined genotype model.

Genotypes	Cases/Person-Years	Age, sex, race-Adjusted Hazard Ratio (95%CI)	P value*
0/1 unfavorable genotypes+	18/569	1.00	
2 unfavorable genotypes	23/424	1.79 (0.96–3.35)	0.07
3 unfavorable genotypes	25/209	4.03 (2.13–7.62)	<0.0001

*P_linear_ _trend_<0.0001.

+ unfavorable genotypes include *EGF* non-AA, *IL28B* non-CC, and *PNPLA3* non-CC.

EGF: Epidermal Growth Factor; IL28B: interleukin-28B; PNPLA3: patatin-like phospholipase domain-containing protein 3; CI: confidence interval.

When we excluded a single subject who developed an outcome within 6 months of the index biopsy, the HRs for the association of the SNPs with risk of clinical deterioration were not materially altered. We performed a sensitivity analysis limiting the outcome to the time to development of ascites, variceal hemorrhage, or HE. *EGF* non-AA genotype remained a significant predictor of increased risk of clinical deterioration (age, sex, race-adjusted HR = 2.93; 95%CI 1.30–6.60; p = 0.01). The association between *PNPLA3* genotype and clinical deterioration also persisted (age, sex, race-adjusted HR = 1.93; 95%CI 1.10–3.37; p = 0.02 for non-CC compared to CC). However, the association between *IL28B* genotype and clinical deterioration was no longer significant (age, sex, race-adjusted HR = 1.52; 95%CI 0.83–2.79; p = 0.18 for non-CC compared to CC). We also conducted analyses restricted to those with Child-Pugh Class A cirrhosis, and found that *EGF* non-AA genotype was associated with the risk of clinical deterioration in Child-Pugh Class A subjects (age, sex, race-adjusted HR = 3.30; 95%CI 1.46–7.35; p = 0.004). The significant association between *PNPLA3* genotype and risk of clinical deterioration also persisted (age, sex, race-adjusted HR = 2.04; 95%CI 1.16–3.60; p = 0.01 for non-CC compared with CC). The association between *IL28B* genotype and risk of clinical deterioration did not remain statistically significant (age, sex, race-adjusted HR = 1.81; 95%CI 0.95–3.48; p = 0.07 for non-CC compared with CC).

One hundred twenty-six patients (75%) received some course of antiviral therapy with either interferon alfa alone or interferon alfa and ribavirin following the index biopsy. Among these, forty patients (32%) achieved a SVR to antiviral therapy. Among these forty patients, only 3 patients developed clinical deterioration. One patient had an episode of variceal hemorrhage and two patients developed HCC. When we incorporated achievement of SVR into the age, sex, and race-adjusted model, *EGF* non-AA genotype remained a significant predictor of increased risk of clinical deterioration (HR = 2.74; 95%CI 1.32–5.67; p = 0.007). There were no longer significant associations between *IL28B* genotype (age, sex, race-adjusted HR 1.61; 95%CI 0.93–2.79; p = 0.09 for non-CC compared with CC) and *PNPLA3* genotype (age, sex, and race-adjusted HR 1.56; 95%CI 0.96–2.56; p = 0.07 for non-CC compared with CC) and clinical deterioration. The addition of SVR to the age, sex, and race-adjusted model with all 3 loci did not materially alter the results.

## Discussion

We found significant associations between *EGF, IL28B*, and *PNPLA3* genotypes and the risk of clinical deterioration in patients with HCV-related cirrhosis. The risk associated with *EGF* genotype persisted, even after adjustment for known clinical predictors of progression, including albumin, bilirubin, and platelets. These results suggest that genetic variation at the *EGF* locus is independently associated with clinical deterioration in patients with CHC and provides prognostic information beyond known clinical predictors. In contrast, genetic variations at *IL28B* and *PNPLA3* loci were associated with prognosis in age, sex, and race-adjusted models, but the association was no longer statistically significant after adjustment for albumin, bilirubin, and platelets. This may reflect either a weaker association of these polymorphisms with clinical deterioration compared with the *EGF* polymorphism or the possibility that the effect of the *IL28B* and *PNPLA3* polymorphisms on clinical deterioration may be mediated by these clinical factors. Thus, controlling for these variables may mask a true biological relation. Our combined analysis shows that the risk of clinical deterioration significantly increases with the presence of each unfavorable genotype and suggests that all three SNPs in concert could be useful in predicting disease progression.

Our findings are consistent with prior data regarding the *EGF* locus and clinical outcome in liver disease. We previously found that the *EGF* AG and GG genotypes are associated with a two to four-fold increased risk for HCC [10]. Moreover, EGF expression, as assessed in a gene expression signature in non-tumoral liver tissue, is associated with poor survival in HCC patients after resection and with progression to advanced cirrhosis, HCC development and poor survival in HCV-related early-stage cirrhosis [20], [21]. Our findings extend the results of these studies by identifying a role for the *EGF* locus independently or in concert with two additional SNPs in not only the development of HCC but also the progression of HCV-related cirrhosis. Consistent with these results is a cross-sectional study showing that the G allele of *EGF* rs4444903 is associated with higher degrees of liver fibrosis in younger subjects with CHC [12].

Our results are consistent with the functional nature of *EGF* rs4444903. We have previously reported increased stability of *EGF* 61*G allele transcripts compared to *EGF* 61*A allele transcripts in human hepatoma cell lines and primary human hepatocytes as well as increased levels of serum and liver EGF in subjects with cirrhosis who have the *EGF* GG versus AA genotype [10]. Additionally, the EGF receptor (EGFR) [22] and its HRas signaling pathway have been identified as host factors for HCV cellular entry [23]. HCV infection also induces EGFR signaling in cell culture models [24] and increases EGFR expression in HCV-infected patients [23]. Taken together with our study, these findings support a key role for EGF in the mediation of CHC-related liver damage.

Our finding of an association between increased risk of clinical deterioration and the *IL28B* non-CC genotype in age, sex, and race-adjusted analysis is supported by evidence that carriage of the T allele is associated with fibrosis severity, cirrhosis and HCC in CHC [7], [9]. In contrast to our results, however, one group showed that the CC genotype was associated with increased risk for adverse clinical outcomes [8]. This group also found no association between *IL28B* genotype and fibrosis progression. The difference in our findings may be related to the fact that their cohort consisted of patients who were prior non-responders to interferon and ribavirin and had advanced fibrosis or cirrhosis. Of note, the association between *IL28B* genotype and clinical deterioration was not significant in our analysis when restricted to Child Pugh Class A subjects or after excluding HCC from the definition of the primary outcome. This may reflect limited power in this smaller cohort and potentially a more limited role for the *IL28B* genotype in hepatic decompensation compared with development of HCC. The association between *IL28B* genotype and clinical deterioration was also no longer significant after adjustment for SVR. This likely reflects the fact that the impact of *IL28B* on clinical deterioration is mediated through response to therapy or potentially limited power to detect the association in the larger model.

Our results showing an association between *PNPLA3* non-CC genotype and risk of clinical deterioration in age, sex, and race-adjusted analysis is supported by a study showing an association between the *PNPLA3* C>G polymorphism and fibrosis progression in CHC [14] and a meta-analysis showing an association between this polymorphism and hepatocarcinogenesis in subjects of European descent [16].

Our study has several limitations. First, we excluded sixteen subjects from the multivariable model adjusting for known predictors of disease progression due to missing laboratory variables. This may have limited our power to detect the association between the *PNPLA3* and *IL28B* polymorphisms and clinical deterioration in analyses adjusted for established laboratory predictors of outcome. Second, we did not have information on disease duration prior to the biopsy. However, no patients had evidence of decompensated liver disease prior to the biopsy and our results were consistent even among those with Child-Pugh Class A disease. Third, because the index biopsies were performed between 1990 and 2007, we did not have reliable information quantifying the amount of steatosis on each biopsy, and thus could not incorporate this into our multivariable model. While steatosis has been shown to predict fibrosis progression prior to the development of cirrhosis, it has not been shown to predict decompensation once cirrhosis has occurred. Fourth, our population was predominantly Caucasian males, limiting the external generalizability of our findings. However, recent meta-analyses revealed that although allele frequencies of the *EGF* polymorphism vary according to race, the association between *EGF* genotype and HCC risk appears independent of race [25], [26]. Nonetheless, further studies are required to validate our findings in more diverse cohorts. Last, we only examined clinical deterioration among individuals with hepatitis C-related cirrhosis. It is unknown if our findings would be similar among patients with other etiologies of cirrhosis. However, a recent study reported an association between carriage of the *EGF* G allele and cirrhosis in 62 subjects with chronic HBV infection [27].

In conclusion, our findings support a role for *EGF* and possibly *IL28B* and *PNPLA3* genotyping in identifying persons with CHC at high risk for disease progression. In light of our group's finding that pharmacological inhibition of EGFR with erlotinib prevented progression of cirrhosis and regressed fibrosis in animal models of progressive cirrhosis [28], EGFR inhibition may be a promising therapeutic approach for reduction of fibrogenesis and prevention of HCC in high risk patients. Taken together with the known association of genetic variation in the *EGF* gene with EGF levels, our data support the potential for *EGF* genotyping to identify patients who may be candidates for strategies to modulate EGF.
